# The genome assemblies of the Tui chub, *Siphateles bicolor*, and Arroyo chub, *Gila orcuttii*

**DOI:** 10.1093/jhered/esag002

**Published:** 2026-01-15

**Authors:** Henry K Baker, Cheyenne Y Payne, Merly Escalona, Jeff Rodzen, Steve Parmenter, Russell M Barabe, Claire Ingel, Mohan P A Marimuthu, Oanh Nguyen, Noravit Chumchim, Eric Beraut, Samuel Sacco, William Seligmann, Colin W Fairbairn, Robert D Cooper, Courtney Miller, Erin Toffelmier, J Carlos Garza, H Bradley Shaffer, Diana J Rennison, Jonathan B Shurin

**Affiliations:** Department of Ecology, Behavior, and Evolution, University of California, San Diego, CA, United States; Institute of Marine Sciences, University of California, Santa Cruz, CA, United States; Southwest Fisheries Science Center, National Marine Fisheries Service, Santa Cruz, CA, United States; Department of Biomolecular Engineering, University of California Santa Cruz, California, United States; Genetics Research Laboratory, California Department of Fish and Wildlife, Sacramento, CA, United States; California Department of Fish and Wildlife, Bishop, CA, United States (Retired); California Department of Fish and Wildlife, Murrieta, CA, United States; California Department of Fish and Wildlife, Sacramento, CA, United States; DNA Technologies and Expression Analysis Core Laboratory, Genome Center, University of California- Davis, CA, United States; DNA Technologies and Expression Analysis Core Laboratory, Genome Center, University of California- Davis, CA, United States; DNA Technologies and Expression Analysis Core Laboratory, Genome Center, University of California- Davis, CA, United States; Department of Ecology and Evolutionary Biology, University of California, Santa Cruz, Santa Cruz, CA, United States; Department of Ecology and Evolutionary Biology, University of California, Santa Cruz, Santa Cruz, CA, United States; Department of Ecology and Evolutionary Biology, University of California, Santa Cruz, Santa Cruz, CA, United States; Department of Ecology and Evolutionary Biology, University of California, Santa Cruz, Santa Cruz, CA, United States; Department of Ecology and Evolutionary Biology, University of California, Los Angeles, CA, United States; Department of Ecology and Evolutionary Biology, University of California, Los Angeles, CA, United States; Department of Ecology and Evolutionary Biology, University of California, Los Angeles, CA, United States; La Kretz Center for California Conservation Science, Institute for Environment and Sustainability, University of California, Los Angeles, CA, United States; Institute of Marine Sciences, University of California, Santa Cruz, CA, United States; Southwest Fisheries Science Center, National Marine Fisheries Service, Santa Cruz, CA, United States; Department of Ocean Sciences, University of California, Santa Cruz, Santa Cruz, CA, United States; Department of Ecology and Evolutionary Biology, University of California, Los Angeles, CA, United States; La Kretz Center for California Conservation Science, Institute for Environment and Sustainability, University of California, Los Angeles, CA, United States; Department of Ecology, Behavior, and Evolution, University of California, San Diego, CA, United States; Department of Ecology, Behavior, and Evolution, University of California, San Diego, CA, United States

**Keywords:** California conservation genomics project, CCGP, conservation genetics, *Cypriniformes*, *Leuciscidae*, freshwater fish

## Abstract

We present genome assemblies for two cyprinoid fishes, the tui chub (*Siphateles bicolor*) and the arroyo chub (*Gila orcuttii*). These fishes are ecologically important representatives of native fish assemblages in the western United States and are both species of conservation concern. The two species hybridize where introductions bring them into contact, with potentially important ecological and evolutionary implications that have not yet been thoroughly examined from a genomic perspective. We present de novo assemblies for both species, representing the first scaffold-level genomes within their respective genera, which were developed as part of the California Conservation Genomics Project using Pacific Biosciences HiFi and Omni-C data. Our tui chub assembly consists of 258 scaffolds spanning 1,148,084,093 base pairs, has a scaffold N50 of 45.9 mb, a contig N50 of 23.7 mb, and a BUSCO completeness score of 98.1%. Our arroyo chub assembly consists of 179 scaffolds spanning 1,263,410,250 base pairs, has a scaffold N50 of 50.5 mb, a contig N50 of 13.1 mb, and a BUSCO completeness score of 97.8%. A comparative analysis of the two species revealed relatively conserved genomes, with the exception of two inversions at chromosome 20. We annotated a total of 34,090 genes with a BUSCO completeness score of 98.1% for the tui chub, and 28,193 genes with a score of 97.4% for the arroyo chub. These assemblies will be valuable resources for characterizing the species’ phylogeographic histories and delineating the role of hybridization in their evolution.

## Introduction

**Fig. 1:**
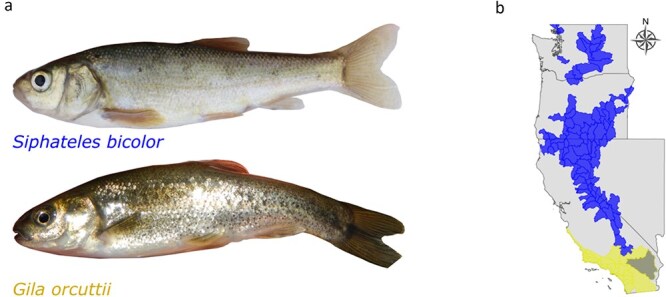
a) Photos of adult tui chub (*Siphateles bicolor*; Little Hot Creek, Mono County, CA) and arroyo chub (*Gila orcuttii*; West Fork San Luis Rey River, San Diego County, CA) and b) a map of their respective distributions in the western United States with *S. bicolor* shown in blue, *G. orcuttii* shown in yellow, and their overlap in dark gray (Photos: H. Baker).

The tui chub (*Siphateles bicolor*) and arroyo chub (*Gila orcuttii*) are closely related cyprinoid fishes (family: *Leuciscidae*; subfamily: *Laviniinae*) of western North America, a region with high endemism and rapid speciation of freshwater fishes (Hubbs and Miller 1948; Smith et al. 2002). Both species belong to the Western Chub-Pikeminnow Clade, which also includes the genera *Mylopharodon* and *Ptychocheilus*, and is characterized by high morphological diversity that is weakly correlated with genetic diversity (Schönhuth et al. 2012; Schönhuth et al. 2018). Arroyo chub are endemic to California and are native to coastal drainages near the city of Los Angeles, and have been introduced to other basins (Moyle 2002). Tui chub are more broadly distributed than arroyo chub, with a native range spanning from the Mojave River basin in southern California to the Columbia River basin in Washington state (Moyle 2002) (Fig. 1). The two species have overlapped in the Mojave River basin (to which arroyo chub were introduced) and have a history of hybridization (Hubbs and Miller 1943), a common phenomenon among western minnows (Hubbs 1955).

Both species are of conservation concern; the arroyo chub has been extirpated from much of its native range, with remaining populations fragmented and limited to headwater reaches in most watersheds where it persists (Benjamin et al. 2016; O’Brien and Barabe 2022). Correspondingly, it is listed as a Species of Special Concern by the state of California and is the target of active conservation efforts (Moyle et al. 2015). The tui chub comprises several subspecies endemic to small isolated basins throughout the arid western United States, many of which are at risk of extinction due to habitat loss, environmental change, and hybridization with introduced taxa. Two subspecies of tui chub are listed as “Endangered” under the US Endangered Species Act: the Mohave tui chub (*S. bicolor mohavensis*), endemic to the exceptionally arid Mojave River basin where it is thought to have been replaced by arroyo chub due to competition and reproductive interference after a brief period of hybridization (Hubbs and Miller 1943; Chen et al. 2013); and the Owens tui chub (*S. bicolor snyderi*), endemic to the Owens River Basin, which has been driven to the verge of extinction as a result of introgressive hybridization with Lahontan tui chub (*S. bicolor obesa*), which were introduced from the adjacent Lahontan Basin (Miller 1973; Chen et al. 2007).

Both species have relatively limited existing genetic information. For arroyo chub, Benjamin et al. (2016) used microsatellite markers to show moderate to high genetic diversity (as measured by mean heterozygosity) and moderate population structure. Microsatellite markers have also been used to characterize genetic diversity in Mohave tui chub (Chen et al. 2013) and Lahontan tui chub (Finger and May 2015), and to characterize introgressive hybridization between Lahontan and Owens tui chubs and distinguish the “toikona tui chub” originating from Cabin Bar Ranch (Chen et al. 2007). The mitochondrial genome of the Mohave tui chub has also been previously assembled (Glaser et al. 2017), and the phylogenetic relationships among putative subspecies of tui chub have been evaluated using RADseq data (Campbell et al. 2025).

Here, we present the first scaffold-level genome assemblies for both the tui chub and arroyo chub, generated as part of the California Conservation Genomics Project (CCGP), a coordinated effort to assemble reference genomes and conduct landscape genomic analyses for more than 150 of California’s native species of conservation concern (Shaffer et al. 2022). These reference genomes will be invaluable resources in studies of phylogenomics and evolution of western cyprinoid fishes, and will inform targeted conservation and management efforts.

## Methods

### Biological materials

For tui chub, we used one adult fish captured on 7 July 2020 in Little Hot Creek (37°41′21.0″N, 118°50′18.8″W) in the Owens River basin near Mammoth Lakes, CA. The individual we sequenced was captured from a population with a documented history of introgression by Lahontan tui chub. We transported the fish live to the Sierra Nevada Aquatic Research Laboratory, where we humanely euthanized and immediately dissected it on ice. For arroyo chub, we captured one adult fish in Tijeras Creek (33°36′45.0″N 117°36′54.0″W) near Rancho Santa Margarita, CA on 5 August 2020 and transported it live to the University of California, San Diego, where we humanely euthanized and dissected it on ice. For both tui and arroyo chub, we dissected samples of several biological materials, including the caudal fin samples that were ultimately used for DNA extraction and sequencing, and several tissue types used for RNA extraction and sequencing, which we flash froze in liquid nitrogen, stored at −80 °C, and shipped on dry ice to the UC Davis Genome Center (Davis, CA) for high molecular weight (HMW) genomic DNA (gDNA) extraction.

#### Nucleic acid library preparation

For each species, HMW gDNA was extracted from caudal fin tissue (34 mg of fin tissue from *G. orcuttii*, specimen ID: AC-1-2; 18 mg of fin tissue from *S. bicolor,* specimen id: 511) using the Nanobind Tissue Big DNA kit (Pacific BioSciences—PacBio, Menlo Park, CA), following the manufacturer’s instructions. We assessed the DNA purity using absorbance ratios (260/280 = 1.83 for both species and 260/230 = 2.38 for *S. bicolor* and 2.48 for *G. orcuttii*) measured using the NanoDrop ND-1000 spectrophotometer. The DNA yield (63 μg total for *G. orcuttii* and 36 μg for *S. bicolor*) was quantified using the Quantus Fluorometer (QuantiFluor ONE dsDNA Dye assay; Promega, Madison, WI). We estimated the size distribution of the HMW DNA using the Femto Pulse system (Agilent, Santa Clara, CA) and found that 83% of the DNA fragments were 100 kb or longer for *G. orcuttii* and 79% for *S. bicolor*.

For both species, HiFi SMRTbell libraries were constructed using the SMRTbell Express Template Prep Kit v2.0 (PacBio, Cat. #100-938-900) according to the manufacturer’s instructions. HMW gDNA was sheared to a target DNA size distribution of 15 to 18 kilo bases (kb) using Diagenode’s Megaruptor 3 system (Diagenode, Belgium; Cat. B06010003). The sheared gDNA was concentrated using 0.45X of AMPure PB beads (PacBio, Cat. #100-265-900) for the removal of single-strand overhangs at 37 °C for 15 min, followed by further enzymatic steps of DNA damage repair at 37 °C for 30 min, end repair and A-tailing at 20 °C for 10 min and 65 °C for 30 min, and ligation of overhang adapters v3 at 20 °C for 60 min. The SMRTbell library was purified and concentrated with 1X AMPure PB beads for nuclease treatment at 37 °C for 30 min and size selection using the BluePippin/PippinHT system (Sage Science, Beverly, MA; Cat #BLF7510/HPE7510) to collect fragments greater than 7 to 9 kb. The 15 to 20 kb average HiFi SMRTbell libraries were sequenced at UC Davis DNA Technologies Core (Davis, CA) using two 8 M SMRT cells, Sequel II sequencing chemistry 2.0, and 30-h movies each on a PacBio Sequel IIe sequencer.

#### Omni-C methods

The Omni-C library was prepared using the Dovetail Omni-C Kit (Dovetail Genomics, Scotts Valley, CA) according to the manufacturer’s protocol with slight modifications. First, specimen tissue (*Gila*: liver, ID: AC-1-8; *Siphateles*: fin, ID: 511-4) was thoroughly ground with a mortar and pestle while cooled with liquid nitrogen. Subsequently, chromatin was fixed in place in the nucleus. The suspended chromatin solution was then passed through 100- and 40-μm cell strainers to remove large debris. Fixed chromatin was digested under various conditions of DNase I until a suitable fragment length distribution of DNA molecules was obtained. Chromatin ends were repaired and ligated to a biotinylated bridge adapter followed by proximity ligation of adapter containing ends. After proximity ligation, cross-links were reversed and the DNA was purified from proteins. Purified DNA was treated to remove biotin that was not internal to ligated fragments. A gDNA library was generated using an NEB Ultra II DNA Library Prep kit (New England Biolabs, Ipswich, MA) with an Illumina compatible y-adaptor. Biotin-containing fragments were then captured using streptavidin beads. The post capture product was split into two replicates prior to PCR enrichment to preserve library complexity with each replicate receiving unique dual indices. The library was sequenced at Vincent J. Coates Genomics Sequencing Lab (Berkeley, CA) on a NovaSeq 6000 platform (Illumina, CA) to generate approximately 100 million 2 × 150 bp read pairs per gigabase (Gb) of genome size.

#### RNA extraction, library preparation, and sequencing

Total RNA was extracted from several tissues (*Siphateles*: caudal fin, a mix of liver/muscle/heart/gonads; *Gila*: heart, gonad, fin, muscle) using a Qiagen RNeasy Mini Kit (Qiagen, Netherlands), according to the manufacturer’s protocol. RNA libraries were then prepared using the KAPA mRNA HyperPrep Kit (Roche, Switzerland) according to the manufacturer’s protocol. Libraries were sequenced with 100 bp reads on an Illumina NovaSeq 6000 platform (Illumina, San Diego, CA), to generate approximately 50 M reads per library.

#### Nuclear genome assembly

We assembled the genomes of the *G. orcuttii* and *S. bicolor* following the CCGP assembly pipeline Version 5.0, as outlined in Table 1, which lists the tools and nondefault parameters used in the assembly process. We removed the remnant adapter sequences from the PacBio HiFi dataset using HiFiAdapterFilt (Sim et al. 2022) and generated the initial phased diploid assemblies using HiFiasm (Cheng et al. 2021; Cheng et al. 2022) on Hi-C mode, with the filtered PacBio HiFi reads and the Omni-C dataset. We then aligned the Omni-C data to each assembly separately following the Arima Genomics Mapping Pipeline (https://github.com/ArimaGenomics/mapping_pipeline) and scaffolded all assemblies with SALSA (Ghurye et al. 2017; Ghurye et al. 2019).

**Table 1:** Assembly pipeline and software used for both *Gila orcuttii* and *Siphateles bicolor*.

**Assembly step**	**Software and any nondefault options**	**Version**	**Reference**
**Initial assembly**			
Filtering PacBio HiFi adapters	HiFiAdapterFilt	Commit 64d1c7b	Sim et al. (2022)
K-mer counting	Meryl (k = 21)	1	https://github.com/marbl/meryl
Estimation of genome size and heterozygosity	GenomeScope (k = 21)	2	Ranallo-Benavidez et al. (2020)
De novo *assembly (contiging)*	HiFiasm (Hi-C Mode, –primary, output hic.hap1.p_ctg, hic.hap2.p_ctg)	0.16.1-r375	Cheng et al. (2021), Cheng et al. (2022)
**Scaffolding**			
Omni-C data alignment	*Arima Genomics Mapping Pipeline*	Commit 2e74ea4	https://github.com/ArimaGenomics/mapping_pipeline
*Arima Genomics Mapping Pipeline (AGMP)*	BWA-MEM	0.7.17-r1188	Li (2013)
	samtools	1.11	Danecek et al. (2021)
	filter_five_end.pl (AGMP)	Commit 2e74ea4	https://github.com/ArimaGenomics/mapping_pipeline
	two_read_bam_combiner.pl (AGMP)	Commit 2e74ea4	https://github.com/ArimaGenomics/mapping_pipeline
	picard	2.27.5	https://broadinstitute.github.io/picard/
**Omni-C contact map generation**			
Short-read alignment	BWA-MEM (-5SP)	0.7.17-r1188	Li (2013)
SAM/BAM processing	samtools	1.11	Danecek et al. (2021)
SAM/BAM filtering	pairtools	0.3.0	Open2C et al. (2024)
Pairs indexing	pairix	0.3.7	Lee et al (2022)
Matrix generation	cooler	0.8.10	Abdennur and Mirny (2020)
Matrix balancing	hicExplorer (hicCorrectmatrix correct --filterThreshold -2 4)	3.6	Ramírez et al. (2018)
Contact map visualization	HiGlass	2.1.11	Kerpedjiev et al. (2018)
	PretextMap	0.1.4	https://github.com/wtsi-hpag/PretextView
	PretextView	0.1.5	https://github.com/wtsi-hpag/PretextMap
	PretextSnapshot	0.0.3	https://github.com/wtsi-hpag/PretextSnapshot
Manual curation tools	Rapid curation pipeline (Wellcome Trust Sanger Institute, Genome Reference Informatics Team)	Commit 7acf220c	https://gitlab.com/wtsi-grit/rapid-curation
**Genome quality assessment**			
Basic assembly metrics	QUAST (--est-ref-size)	5.0.2	Gurevich et al. (2013)
Assembly completeness	BUSCO (-m geno, -l actinopterygii)	5.0.0	Manni et al. (2021)
	Merqury	29 January 2020	Rhie et al. (2020)
**Contamination screening**			
Local alignment tool	BLAST+ (-db nt, -outfmt '6 qseqid staxids bitscore std' , -max_target_seqs 1, -max_hsps 1, -evalue 1e-25)	2.15	Camacho et al. (2009)
General contamination screening	BlobToolKit (HiFi coverage, BUSCO = actinopterygii, NCBI Taxa ID = 71773 for *Siphateles bicolor*and 71755 for *Gila orcutti*)	2.3.3	Challis et al. (2020)
**Interspecific genome comparisons**			
Synteny evaluation	CHROMEISTER		Pérez-Wohlfeil et al. (2019)
Genome alignment and filtering	MUMmer4 (nucmer -l 100 -c 200; delta-filter -l 1,000 -q -r)	4.0.0	Marçais et al. (2018)

Software citations are listed in the text.

We manually curated the assemblies for both species by iteratively generating and analyzing their corresponding Omni-C contact maps. To generate the contact maps, we aligned the Omni-C data with BWA-MEM (Li 2013), identified ligation junctions, and generated Omni-C pairs using pairtools (Open2C et al. 2024). We generated a multiresolution Omni-C matrix with cooler (Abdennur and Mirny 2020) and balanced it with hicExplorer (Ramírez et al. 2018). We used HiGlass (Kerpedjiev et al. 2018) and the PretextSuite (https://github.com/wtsi-hpag/PretextView;  https://github.com/wtsi-hpag/PretextMap;  https://github.com/wtsi-hpag/PretextSnapshot) to visualize the contact maps where we identified misassemblies and misjoins, and finally modified the assemblies using the Rapid Curation pipeline from the Wellcome Trust Sanger Institute, Genome Reference Informatics Team (https://gitlab.com/wtsi-grit/rapid-curation). Some of the remaining gaps (joins generated during scaffolding and/or curation) were closed using the PacBio HiFi reads and YAGCloser (https://github.com/merlyescalona/yagcloser). Finally, we checked for contamination using the BlobToolKit Framework (Challis et al. 2020).

### Genome quality assessment

We generated k-mer counts from the PacBio HiFi reads using meryl (https://github.com/marbl/meryl). The k-mer counts were then used in GenomeScope2.0 (Ranallo-Benavidez et al. 2020) to estimate genome features including genome size, heterozygosity, and repeat content. To obtain general contiguity metrics, we ran QUAST (Gurevich et al. 2013). To evaluate genome quality and functional completeness, we used BUSCO (Manni et al. 2021) with the Actinopterygii ortholog database (actinopterygii_odb10) which contains 3,640 genes. Assessment of base level accuracy (quality value [QV]) and k-mer completeness was performed using the previously generated meryl database and merqury (Rhie et al. 2020). We further estimated genome assembly accuracy via BUSCO gene set frameshift analysis using the pipeline described by Korlach et al. (2017). Measurements of the size of the phased blocks are based on the size of the contigs generated by HiFiasm on Hi-C mode. We follow the quality metric nomenclature established by Rhie et al. (2021), with the genome quality code x.y.P.Q.C, where, x = log10[contig NG50]; y = log10[scaffold NG50]; P = log10 [phased block NG50]; Q = Phred base accuracy QV; C = % genome represented by the first “n” scaffolds, following a karyotype of 2n = 50, an estimated value of the number of chromosomes for this species based on the mode of the number of chromosome from other closely related species (Genome on a Tree—GoaT; tax_tree(*G. orcuttii* or *S. bicolor*); Challis et al. 2023). Quality metrics for the notation were calculated on the haplotype one assembly.

### Genome annotation

We annotated the reference assembly for each species using the NCBI Eukaryotic Genome Annotation Pipeline v0.3.2-alpha (hereafter, “EGAPx”) which is published in the National Center for Biotechnology Information (NCBI) RefSeq database (O’Leary et al. 2016) and accessible through the NCBI github page (“ncbi/egapx”). Annotation features were identified by aligning transcripts and proteins from related taxa in the RefSeq database using BLAST (Camacho et al. 2009). Novel, species-specific RNA-Seq reads generated from five tissue types were also aligned to the assembly using the alignment software STAR (Dobin et al. 2013). Additional features are predicted using Hidden Markov-based gene models using the NCBI Gnomon software. We evaluated the quality and completeness of our annotation by comparing the longest protein for each annotated coding gene to eukaryotes (odb12) and Actinopterygii (odb12) using BUSCO v5.8.2 (Manni et al. 2021). We report the number of annotation features and the BUSCO results in Tables 2 and 3.

**Table 2:** Sequencing and assembly statistics, and accession numbers for *Gila orcuttii*.

Bio projects and vouchers	CCGP NCBI BioProject			PRJNA720569			
	Genera NCBI BioProject			PRJNA765833			
	Species NCBI BioProject			PRJNA808340			
	NCBI Genome BioSample			SAMN31536034			
	NCBI RNA BioSamples			SAMN40863714, SAMN41792251, SAMN41792250, SAMN41792252			
	Specimen identification			AC-1			
	NCBI genome accessions			**Haplotype 1**		**Haplotype 2**	
	Assembly accession			JAPDVR00000000		JAPDVS00000000	
	Genome sequences			GCA_026230005.1		GCA_026230025.1	
Genome sequence	PacBio HiFi reads		Run	1 PACBIO_SMRT (Sequel II) run: 4.1 M spots, 62.8G bases, 39.6G bytes			
			Accession	SRX19355850			
	Omni-C Illumina reads		Run	2 ILLUMINA (Illumina NovaSeq 6000) runs: 367.9 M spots, 111.1G bases, 36.1G bytes			
			Accession	SRX19355851-2			
Genome assembly	Assembly identifier (quality code*)			fGilOrc1(7.7.P7.Q62.C98)			
	HiFi Read coverage^a^			51.29X			
quality metrics				**Haplotype 1**		**Haplotype 2**	
	Number of contigs			506		659	
	Contig N50 (bp)			13,138,541		14,561,125	
	Contig NG50^a^			13,429,114		14,910,512	
	Longest contigs			35,567,425		39,057,877	
	Number of scaffolds			179		341	
	Scaffold N50			50,480,577		49,135,603	
	Scaffold NG50^a^			50,480,577		49,135,603	
	Largest scaffold			75,862,728		76,844,234	
	Size of final assembly			1,263,410,250		1,272,848,069	
	Phased block NG50^a^			13,933,662		15,310,579	
	Gaps per Gbp (# Gaps)			259 (327)		250 (318)	
	Indel QV (Frame shift)			52.68970905		51.49437034	
	Base pair QV			62.3386		62.4377	
				Full assembly = 62.388			
	k-mer completeness			94.9712		94.9698	
				Full assembly = 98.9193			
	BUSCO completeness^b^ (actinopterygii) *n* = 3,640		**C**	**S**	**D**	**F**	**M**
		H1^c^	97.80%	96.50%	1.30%	0.80%	1.40%
		H2^c^	97.70%	96.20%	1.50%	0.70%	1.60%
Genome annotation quality metrics				**Count of features**			
	Genes			34,090			
	Transcripts			47,822			
	mRNA			37,414			
	lncRNA			10,267			
	CDSs			37,420			
	BUSCO completeness^b^		**C**	**S**	**D**	**F**	**M**
	Eukaryota_odb12		98.4%	97.7%	0.8%	0.8%	0.8%
	actinopterygii_odb12		98.1%	97.2%	0.9%	0.7%	1.2%

^a^Read coverage and NGx statistics have been calculated based on the estimated genome size of 1.22 Gb

^b^BUSCO Scores. Complete BUSCOs (C). Complete and single-copy BUSCOs (S). Complete and duplicated BUSCOs (D). Fragmented BUSCOs (F). Missing BUSCOs (M).

^c^(H1) Haplotype 1 and (H2) Haplotype 2 assembly values.

*Assembly quality code x.y.P.Q.C derived notation, from (Rhie et al. 2021). x = log10[contig NG50]; y = log10[scaffold NG50]; P = log10 [phased block NG50]; Q = Phred base accuracy QV (Quality value); C = % genome represented by the first ‘n’ scaffolds, following an estimated karyotype for this species of 2n=50 as it is stated in the Genome on a Tree database (Challis et al. 2024). Quality code for all the assembly denoted by primary haplotype assembly (bSipBic1.0.p)

**Table 3:** Sequencing and assembly statistics, and accession numbers for *Siphateles bicolor*.

Bio projects and vouchers	CCGP NCBI BioProject			PRJNA720569			
	Genera NCBI BioProject			PRJNA765862			
	Species NCBI BioProject			PRJNA808376			
	NCBI Genome BioSample			SAMN40933556			
	NCBI RNA BioSample			SAMN41792253, SAMN41792254			
	Specimen identification			CCGP_62_JS_511			
	NCBI genome accessions			**Haplotype 1**		**Haplotype 2**	
	Assembly accession			JBECYN000000000		JBECYM000000000	
	Genome sequences			GCA_042767315.1		GCA_042767305.1	
Genome sequence	PacBio HiFi reads		Run	1 PACBIO_SMRT (Sequel II) run: 4.9 M spots, 70.8G bases, 42.4G bytes			
			Accession	SRX26376895			
	Omni-C Illumina reads		Run	2 ILLUMINA (Illumina NovaSeq 6000) runs: 325.2 M spots, 98.2G bases, 32.5G bytes			
			Accession	SRX26376896-7			
Genome assembly quality metrics	Assembly identifier (Quality code^a^)				fSipBic1(7.7.P7.Q.C98)		
	HiFi Read coverage^b^			64.64X			
				**Haplotype 1**		**Haplotype 2**	
	Number of contigs			351		236	
	Contig N50 (bp)			23,738,801		20,971,927	
	Contig NG50^b^			24,077,237		21,256,759	
	Longest contigs			39,436,206		41,162,026	
	Number of scaffolds			258		141	
	Scaffold N50			45,866,664		45,482,732	
	Scaffold NG50^b^			46,420,387		45,821,946	
	Largest scaffold			70,637,389		69,621,301	
	Size of final assembly			1,148,084,093		1,144,888,142	
	Phased block NG50^b^			24,173,431		20,111,168	
	Gaps per Gbp (# gaps)			81 (93)		83 (95)	
	Indel QV (frame shift)			50.12668534		50.10286385	
	Base pair QV			63.7799		63.7799	
				Full assembly = 63.8293			
	k-mer completeness			90.396		90.3635	
				Full assembly = 99.0723			
	BUSCO completeness^c^ (actinopterygii) *n* = 3,640		**C**	**S**	**D**	**F**	**M**
		H1^d^	98.19%	96.70%	1.40%	0.40%	1.50%
		H2^d^	98.00%	96.50%	1.50%	0.50%	1.50%
Genome annotation quality metrics				**Count of features**			
	Genes			28,193			
	Transcripts			32,628			
	mRNA			27,140			
	lncRNA			5,459			
	CDSs			27,145			
	BUSCO completeness^c^		**C**	**S**	**D**	**F**	**M**
	eukaryota_odb12		98.4%	97.7%	0.8%	0.8%	0.8%
	actinopterygii_odb12		97.4%	96.6%	0.8%	1.0%	1.6%

^a^Assembly quality code x.y.P.Q.C derived notation, from (Rhie et al. 2021). x = log10[contig NG50]; y = log10[scaffold NG50]; P = log10 [phased block NG50]; Q = Phred base accuracy QV (quality value); C = % genome represented by the first “n” scaffolds, following a known karyotype for this species of 2n = 50 (Genome on a Tree, query (*Siphateles bicolor*); Challis et al. 2023). Quality code for all the assembly denoted by haplotype 1 assembly (bSipBic1.0.p)

^b^Read coverage and NGx statistics have been calculated based on the estimated genome size of 1.09 Gb

^c^BUSCO Scores. Complete BUSCOs (C). Complete and single-copy BUSCOs (S). Complete and duplicated BUSCOs (D). Fragmented BUSCOs (F). Missing BUSCOs (M).

^d^(H1)Haplotype 1 and (H2) Haplotype 2 assembly values.

#### Interspecific genome assembly comparison

To evaluate synteny between the genomes of these species, we aligned the 25 chromosome-length scaffolds of each genome assembly with MUMmer4 nucmer https://www.zotero.org/google-docs/?eTzrWU(Marçais et al. 2018; see Table 1 for parameters). We used the MUMmer4 delta-filter function to filter for only the best hit alignments with a length of at least 1kb. Using code modified from jmonlong.github.io, we further filtered these alignments to visualize alignments longer than 10 kb, as well as shorter alignments within 1 kb of these long alignments, with ggplot2::ggplot https://www.zotero.org/google-docs/?LcB8vS (Wickham, 2016https://www.zotero.org/google-docs/?LcB8vS) in R (R Core Team 2023).

## Results

### G. orcuttii

#### Sequencing data

**Fig. 2:**
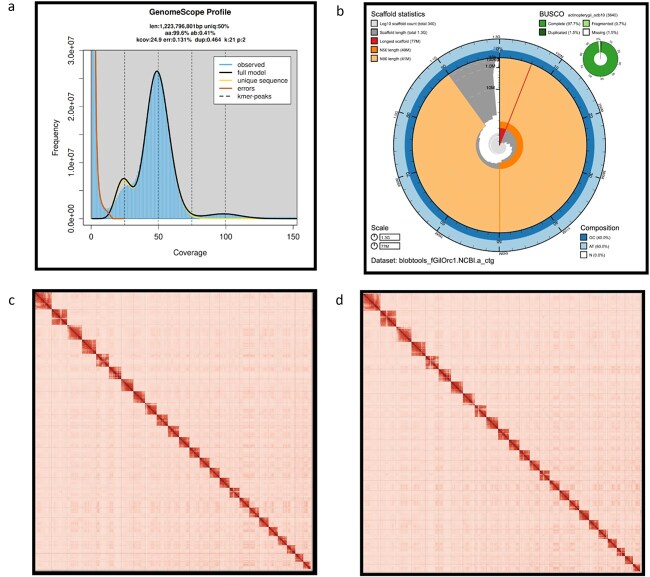
Visual overview of genome assembly metrics for the *Gila orcuttii* haplotype 1 assembly (fGilOrc1). a) Kmer spectrum output generated from PacBio HiFi data without adapters using GenomeScope 2.0. The observed distribution is characteristic of an outbred diploid genome, with bimodality indicating presence of heterozygosity. b) BlobToolKit Snailplot showing a graphical representation of the quality metrics outlined in Table 2 for the haplotype 1 *G. orcuttii* assembly and BUSCO scores for the set of orthologues. The plot circle indicates the full size of the assembly. Moving from the center outwards, the central plot depicts length-related metrics. The red line denotes the size of the longest scaffold. All other scaffolds are arranged in size-order moving clockwise around the plot and drawn in gray starting from the outside of the central plot. The dark and light orange arcs highlight the scaffold N50 and scaffold N90 values. The central light gray spiral indicates the cumulative scaffold count with a white line at each order of magnitude. White regions in this area reflect the proportion of Ns in the assembly; the dark versus light blue area around it depict the mean, maximum, and minimum GC vs. AT content at 0.1% intervals (Challis et al. 2020). Omni-C contact Maps for the haplotype 1 (c) and haplotype 2 (d) genome assembly generated using PretextSnapshot. These contact maps translate proximity of genomic regions in 3D space to contiguous linear organization. Each cell in the contact map corresponds to sequencing data supporting the linkage between two of such regions.

The Omni-C and PacBio HiFi sequencing libraries generated 367.88 million read pairs and 4.1 million reads, respectively, prior to filtering. The latter yielded 43.95-fold coverage, with an N50 read length of 15 786 bp, minimum read length of 174 bp, mean read length of 15,276 bp, and maximum read length of 58,041 bp (see read length distribution in Supplementary Fig. S1a). Based on PacBio HiFi long reads, we estimated with Genomescope 2.0 a genome size of 1.22 Gb, 0.131% sequencing error rate, and 0.41% nucleotide heterozygosity. The k-mer spectrum shows a bimodal distribution with two major peaks at 26- and 51-fold coverage (Fig. 2a). Sequencing of mRNA libraries for SAMN40863714, SAMN41792251, SAMN41792250, and SAMN41792252 yielded 48 , 63, 40 , and 47 M paired end reads, respectively.

#### Nuclear genome assembly

The final assembly (fGilOrc1) consisted of two phased haplotypes that vary slightly in size compared with the estimated value from GenomeScope2.0 (Fig. 2a), as has been observed in other taxa (see Pflug et al. 2020 for example). The assemblies are referenced hereafter as haplotype 1 and haplotype 2 based on metrics of completeness; the assembly with better overall metrics being haplotype 1. The haplotype 1 assembly (fGilOrc1.0.hap1) consisted of 179 scaffolds spanning 1.26 Gb with contig N50 of 13.13 mb, scaffold N50 of 50.48 mb, longest contig of 35.56 mb, and largest scaffold of 75.86 mb. The haplotype 2 assembly (fGilOrc1.0.hap2) consisted of 341 scaffolds, spanning 1.27 Gb with contig N50 of 14.5 mb, scaffold N50 of 49.13 mb, largest contig of 39.05 mb, and largest scaffold of 76.84 mb.

During manual curation, we generated a total of 262 joins and 39 breaks; 125 joins and 23 breaks were done on the haplotype 1 assembly, and 137 joins and 16 breaks carried out on haplotype 2. We were able to close a total of 60 gaps, 23 on the haplotype 1 assembly and 37 on the haplotype 2 assembly. We further filtered out five contigs corresponding to undefined virus contaminants (three contigs from the haplotype 1 assembly and two from the haplotype 2 assembly). No further contigs were removed.

The haplotype 1 assembly had a BUSCO completeness score of 97.8% using the Actinopterygii gene set, a per base quality (QV) of 62.33, a kmer completeness of 94.97, and a frameshift indel QV of 52.68. The haplotype 2 assembly had a BUSCO completeness score of 97.7% using the same gene set, a per base quality (QV) of 62.43, a kmer completeness of 94.96, and a frameshift indel QV of 51.49. Assembly statistics are reported in Table 2, and graphical representation for the haplotype 1 assembly in Fig. 2b. The Omni-C contact map showed that both assemblies are highly contiguous with some chromosome-length scaffolds, suggesting the *G. orcutti* genome is organized in 25 chromosomes, based on the number of large bins along the diagonal in the contact maps (Fig. 2c and d). We have deposited scaffolds corresponding to the assemblies of both haplotypes in GenBank (See Table 2 and Data availability for details).

Our final genome annotation for *Gila orcutti* included 34,090 genes, with an Actinopterygii BUSCO completeness of 98.1%. A list of annotation statistics and BUSCO score breakdowns for eukaryotes and Actinopterygii is reported in Table 2.

### S. bicolor

#### Sequencing data

**Fig. 3:**
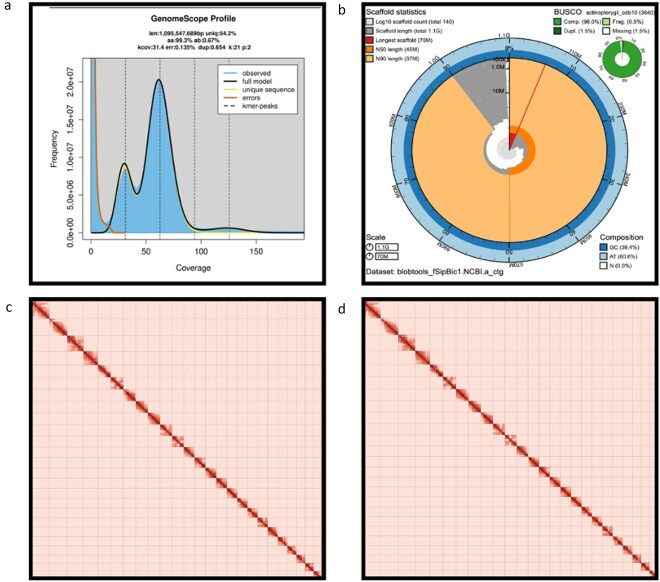
Visual overview of genome assembly metrics for the *Siphateles bicolor* haplotype 1 assembly (fSlipBic1). a) Kmer spectrum output generated from PacBio HiFi data without adapters using GenomeScope 2.0. The observed distribution is characteristic of an outbred diploid genome, with bimodality indicating presence of heterozygosity. b) BlobToolKit Snailplot showing a graphical representation of the quality metrics outlined in Table 3 for the haplotype 1 *S. bicolor* assembly and BUSCO scores for the set of orthologues. The plot circle indicates the full size of the assembly. Moving from the center outwards, the central plot depicts length-related metrics. The red line denotes the size of the longest scaffold. All other scaffolds are arranged in size-order, moving clockwise around the plot and drawn in gray starting from the outside of the central plot. The dark and light orange arcs highlight the scaffold N50 and scaffold N90 values. The central light gray spiral indicates the cumulative scaffold count with a white line at each order of magnitude. White regions in this area reflect the proportion of Ns in the assembly; the dark versus light blue area around it depict the mean, maximum, and minimum GC vs. AT content at 0.1% intervals (Challis et al. 2020). Omni-C contact Maps for the haplotype 1 (c) and haplotype 2 (d) genome assembly generated using PretextSnapshot. These contact maps translate proximity of genomic regions in 3D space to contiguous linear organization. Each cell in the contact map corresponds to sequencing data supporting the linkage between two of such regions.

The Omni-C library generated 202.93 million read pairs and the PacBio HiFi library generated 4.92 million reads. The PacBio HiFi sequences yielded ~64X genome coverage and had an N50 read length of 14,818 bp; a minimum read length of 134 bp; a mean read length of 14,382 bp; and a maximum read length of 55,897 bp (see read length distribution in Supplementary Fig. S1b). Based on the PacBio HiFi data, Genomescope 2.0 estimated a genome size of 1.09 Gb, a 0.135% sequencing error rate, and 0.67% heterozygosity. The k-mer spectrum shows a bimodal distribution with a major peak at ~60-fold coverage and a minor peak at ~31-fold coverage (Fig. 3a). Sequencing of mRNA libraries for SAMN41792253 and SAMN41792254 yielded 41 and 38 M paired end reads, respectively.

#### Nuclear genome assembly

The final genome assembly (fSipBic1) consists of two phased haplotypes, haplotype 1 and haplotype 2, with haplotype 1 being the more complete and contiguous of the two. Both assemblies are similar in size, but not equal to the estimated genome size from GenomeScope2.0, as has been observed in other taxa (see Pflug et al. 2020, for example).

The haplotype 1 assembly (fSlipBic1.0.p) consisted of 258 scaffolds spanning 1.14 Gb with a contig N50 of 23.84 mb, a scaffold N50 of 45.86 mb, the largest contig size of 39.43 mb, and the largest scaffold size of 70.63 mb. The haplotype 2 assembly (fSlipBic1.0.a) consisted of 141 scaffolds spanning 1.14 Gb with a contig N50 of 20.97 mb, a scaffold N50 of 45.48 mb, the largest contig size of 41.16 mb, and the largest scaffold size of 69.62 mb.

The haplotype 1 assembly had a BUSCO completeness score for the Actinopterygii gene set of 98.1%, a base pair QV of 63.87, a kmer completeness of 90.39, and a frameshift indel QV of 50.12. The haplotype 2 assembly had a BUSCO completeness score for the Actinopterygii gene set of 98.0%, a base pair QV of 63.77, a kmer completeness of 90.36%, and a frameshift indel QV of 50.1.

During manual curation, we made a total of 166 joins (82 on haplotype 1 and 84 on haplotype 2) and 33 breaks (13 on haplotype 1 and 20 on the haplotype 2) based on the Omni-C contact map signal. We closed a total of 40 gaps (17 on haplotype 1 and 23 on the haplotype 2). No other contigs were removed or modified. The Omni-C contact maps showed highly contiguous assemblies, with chromosome-length scaffolds, suggesting the genome of *S. bicolor* is organized in 25 chromosomes based on the number of large bins along the diagonal in the contact maps (Fig. 3b). Assembly statistics are reported in Table 2 and represented graphically in Fig. 3c. We have deposited the genome assemblies corresponding to both haplotypes in GenBank (see Table 3 and Data Availability for details).

Our final genome annotation for *S. bicolor* included 28,193 genes and an Actinopterygii BUSCO completeness of 97.4%. The lower gene count for tui chub relative to arroyo chub likely reflects differences in the number of tissue types sequenced. A list of annotation statistics and BUSCO score breakdowns for eukaryotes and Actinopterygii is reported in Table 3.

#### Interspecific genome comparison

**Fig. 4:**
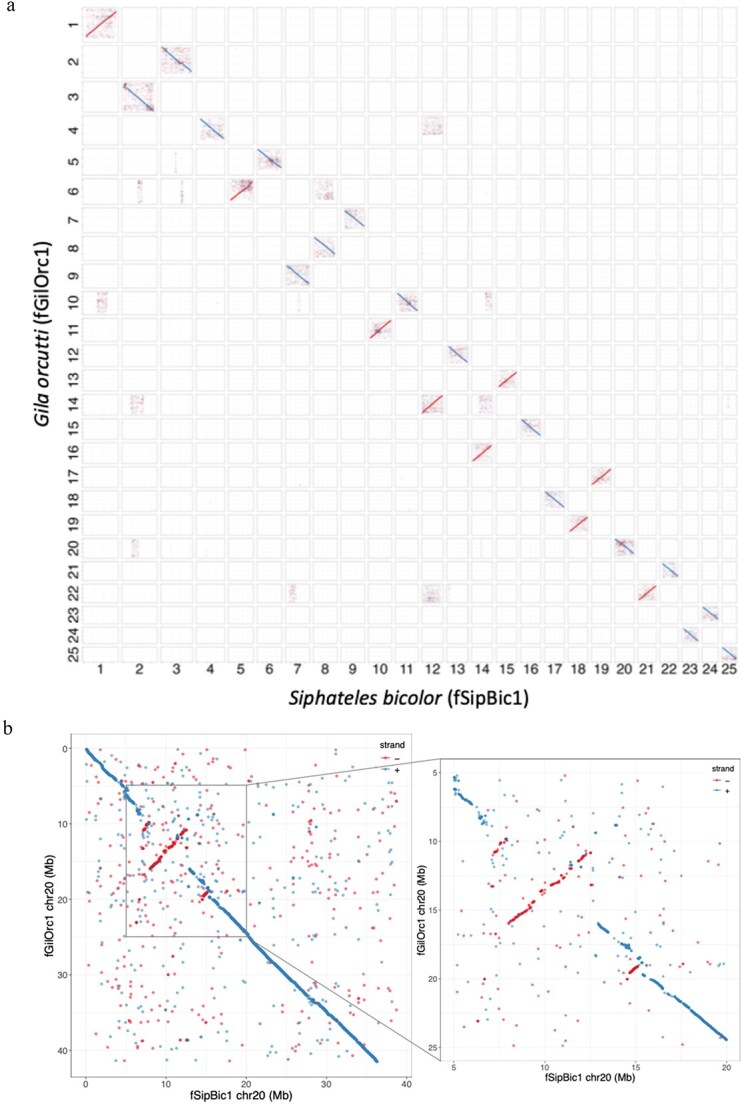
Comparison of the *Siphateles bicolor* and *Gila orcuttii* genome assemblies. a) Alignment dot plots of the 25 chromosome-level scaffolds from each genome assembly, indicating general collinearity between species. b) Alignment of chromosome 20 in both assemblies, with a close-up of a ~5 mb inversion and a smaller downstream ~ 0.5 mb inversion. Blue points indicate a forward alignment and red points indicate a reverse alignment.

We find that the 25 chromosome-length scaffolds of these genomes are generally collinear, with the exception of two inversions on chromosome 20 of both species (Fig. 4). The larger inversion is ~5 mb (fGilOrc1 ~ 11.0 to 16.0 mb and fSipBic1 ~ 8.0 to 12.5 mb) and is followed by a smaller ~0.5 mb inversion (fGilOrc1 ~ 19.1 to 19.6 mb and fSipBic1 ~ 14.6 to 15.1 mb). Chromosome 20 is otherwise syntenic between these species.

## Discussion

The draft reference genomes for the arroyo chub and tui chub represent an important advancement in the evolutionary understanding and conservation of these native California fishes. As freshwater ecosystems face mounting threats from habitat loss, climate change, and introduced species, high resolution genomic data serve as an essential foundation for informed management and recovery strategies. This is particularly true for our focal taxa in California, where growing anthropogenic water demand and increased drought frequency have severely shifted the selective landscape for these fish species, and concurrently, introductions of nonnative taxa have driven hybridization events that have reshuffled genomes and altered phenotype-environment relationships (Hubbs 1955; Miller 1973; Chen et al. 2007; Baker et al. 2021). These draft genomes will be an important resource for assessing genetic diversity, population structure, patterns of introgression, and local adaptation, factors crucial for maintaining resilient populations and guiding effective conservation interventions.

The genomes presented here represent valuable resources for biodiversity and conservation of native fishes. First, they enable fine-scale detection of genetic bottlenecks and inbreeding in fragmented or isolated populations, a common concern in California where chub habitats are increasingly disturbed. For the tui chub, which inhabits a patchily distributed range, the genome will support efforts to resolve taxonomic uncertainty among many subspecies, and will aid efforts to identify evolutionarily significant units (ESUs). With whole-genome data, conservation efforts can move beyond traditional markers (e.g. microsatellites or mitochondrial DNA) to leverage genome-wide scans for signs of selection and adaptive divergence. These data will be crucial for informing reintroduction and habitat restoration programs with a previously unattainable level of precision.

Currently, reference genomes are publicly available for 40 other species within *Leuciscidae*, a diverse family of minnows from North America, Europe, and Asia, including three from the subfamily *Laviniinae* which contains *Siphateles* and *Gila*. This is the first reference genome available for *Siphateles*, and only one other reference genome exists for a *Gila* species, but it is a contig-level assembly (*Gila robusta*; Suchocki et al. 2023). Our assemblies revealed genome sizes of 1.15 Gb and 1.26 Gb for *S. bicolor* and *G. orcuttii*, respectively, which are similar to other reference genomes within *Leuciscidae* (mean = 1.01 Gb, range: 0.62 to 1.32 Gb). These new genomes have already offered the opportunity to describe structural variation between taxa with varying degrees of divergence within *Leuciscidae*. Here, we find that the *S. bicolor* and *G. orcuttii* genomes are generally collinear, with the exception of a ~5 mb and a smaller ~ 0.5 mb inverted region on their otherwise syntenic chromosome 20. Given that these species are known to naturally hybridize when they come in contact (Hubbs and Miller 1943; Greenfield and Greenfield 1972), this region could underlie reduced fitness of hybrids, as was predicted by Chen et al. (2013). Future work can take advantage of these genomes to explore intra- and interspecific variation as well as the genomic landscape of hybrid genomes.

Importantly, these draft genomes bridge a key phylogenetic and biogeographic gap in cyprinid genomic resources, enriching comparative studies across North American freshwater fishes. Sequencing of the arroyo and tui chub genomes ensures that the evolutionary narratives of western North American fishes are more fully represented, which enhances our ability to protect them in a rapidly changing world.

## Supplementary Material

esag002_Supplemental_Figures

## Data Availability

For *Gila orcuttii*, the data generated for this study are available under NCBI BioProject PRJNA808340. Raw sequencing data for sample AC-1 (NCBI BioSample SAMN31536034) are deposited in the NCBI Short Read Archive (SRA) under SRR23446490 for PacBio HiFi sequencing data, and SRR23446488-89 for the Omni-C Illumina sequencing data. GenBank accessions for both haplotype 1 and haplotype 2 assemblies are GCA_026230005.1 and GCA_026230025.1; and for genome sequences JAPDVR000000000 and JAPDVS000000000. Data generated for the annotation in this study are available under NCBI BioProject PRJNA1011933. Raw RNA sequencing data (NCBI BioSamples SAMN40863714, SAMN41792251, SAMN41792250, SAMN41792252) are deposited in the NCBI SRA under accession numbers SRR29377028, SRR29377029, SRR29377030, SRR29377027, respectively. For *Siphateles bicolor*, the data generated for this study are available under NCBI BioProject PRJNA808376. Raw sequencing data for sample CCGP_62_JS_511 (NCBI BioSample SAMN40933556) are deposited in the NCBI Short Read Archive (SRA) under SRR30986051 for PacBio HiFi sequencing data, and SRR30986049, SRR30986050 for the Omni-C Illumina sequencing data. GenBank accessions for both haplotype 1 and haplotype 2 assemblies are GCA_042767315.1 and GCA_042767305.1; and for genome sequences JBECYN000000000 and JBECYM000000000. Data generated for the annotation in this study are available under NCBI BioProject PRJNA1011934. Raw RNA sequencing data (NCBI BioSamples SAMN41792253, SAMN41792254) are deposited in the NCBI SRA under accession numbers SRR29377070, SRR29377069, respectively. For both species, the assembly scripts and other data for the analyses presented can be found at the following GitHub repository: www.github.com/ccgproject/ccgp_assembly.
