# Supplementary material for: The genome assemblies of the Tui chub, Siphateles bicolor, and Arroyo chub, Gila orcuttii
Source: J Hered. 2026 Jan 15;117(4):843–55. doi: 10.1093/jhered/esag002 (PMC13326408; doi:10.1093/jhered/esag002)
Supplement: esag002_Supplemental_Figures [file esag002_supplemental_figures.docx]

***Supplemental Figures:***

| A | 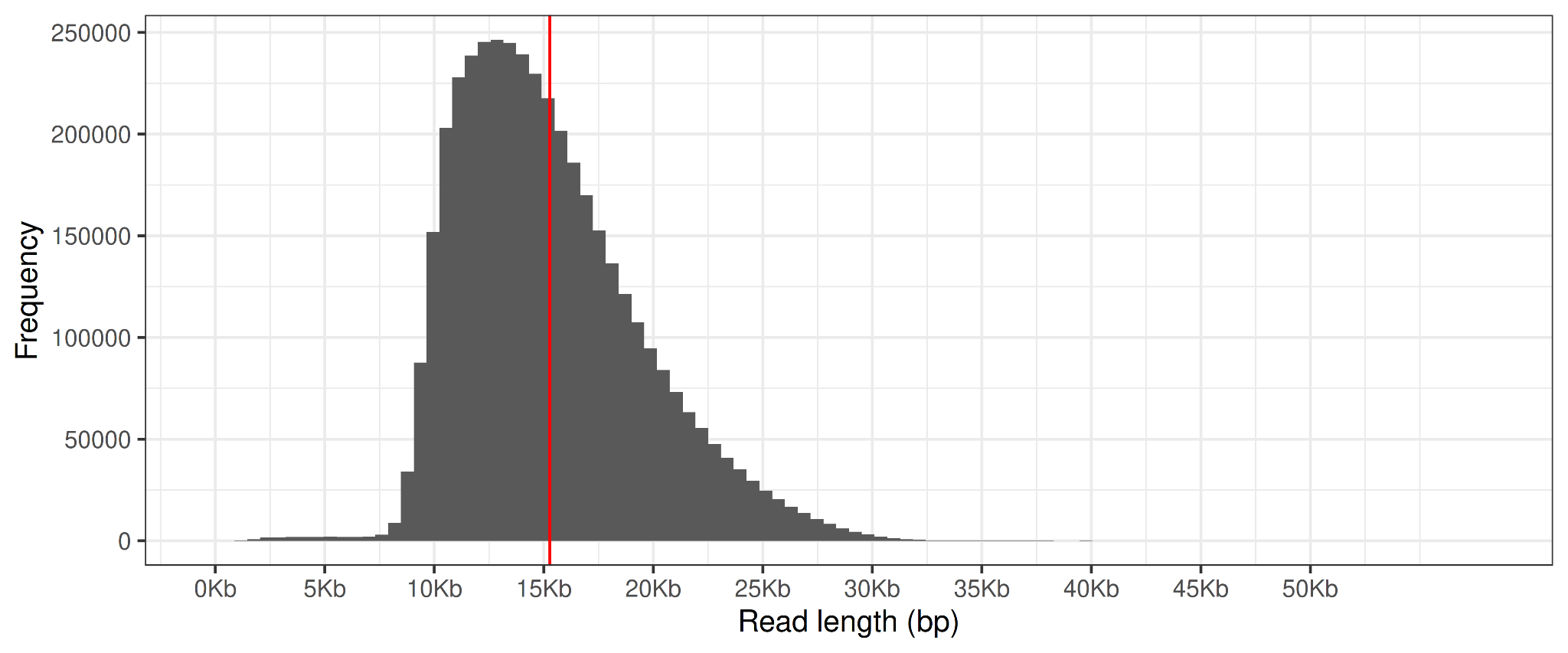 |
| --- | --- |
| B | 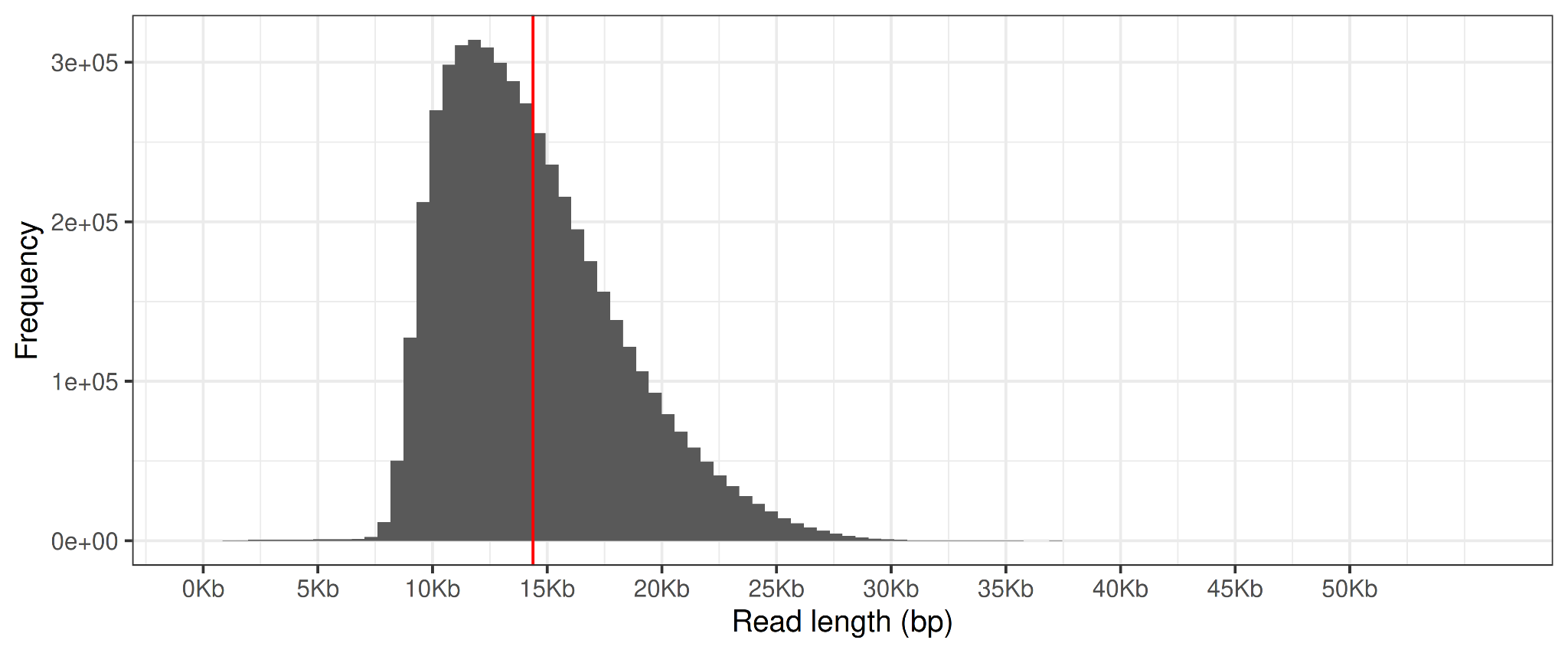 |

S1. Read length distributions for (A) arroyo chub (*G. orcuttii*) and (B) tui chub (*S. bicolor*) (mean shown in red).
